# The urban wage premium is disappearing in U.S. micropolitan areas

**DOI:** 10.1371/journal.pone.0267210

**Published:** 2022-04-14

**Authors:** Shade T. Shutters, J. M. Applegate

**Affiliations:** 1 School of Complex Adaptive Systems, Arizona State University, Tempe, Arizona, United States of America; 2 Global Climate Forum, Berlin, Germany; Szechenyi Istvan University: Szechenyi Istvan Egyetem, HUNGARY

## Abstract

A key driver of urbanization is the pursuit of economic opportunities in cities. One such opportunity is the promise of higher wages in larger cities, a phenomenon known as the urban wage premium. While an urban wage premium has been well-documented among U.S. metropolitan areas, little is known about its existence in micropolitan areas, which represent an important link between rural and dense urban areas. Here we measure the power law scaling coefficient of annual wages versus employment for both U.S. metropolitan and micropolitan areas over a 37-year period. We take this coefficient to be a quantification of the urban wage premium for each type of urban area and find the relationship is superlinear in all years for both area types. Though both area types once had wage premiums of similar magnitude, the wage premium in micropolitan areas has steadily declined since the late 1980s while in metropolitan areas it has generally increased. This growing gap between micropolitan and metropolitan wage premiums is ongoing in parallel to other diverging characteristics, such as inequality and voting behavior, suggesting that our result is part of a broader social, cultural, and political divergence between small and large cities. Finally, we speculate that if urban residents respond to the COVID-19 crisis by migrating, the trends we describe may change significantly.

## Introduction

Globally, more than 3 million people migrate to cities each week–primarily in search of economic opportunities [1]. Those opportunities are partly driven by the existence of a phenomenon known as the urban wage premium. An urban wage premium exists when workers in larger cities earn higher average wages than workers in smaller cities [2–4]. In both the U.S. [5] and Sweden [6] a wage premium has been shown to follow a power-law relationship that scales superlinearly with city size. In other words, workers in larger cities not only earn higher average wages, they do so systematically as a power law function of city size. Bettencourt [7] demonstrated theoretically not only that a wage premium should manifest as a power law function, but predicted the value of its exponent.

Past studies of the U.S. urban wage premium focus almost exclusively on metropolitan statistical areas, which are defined as urbanized areas of at least 50,000 people. Little attention has been paid to the nearly 550 micropolitan statistical areas [8,9], which are urbanized areas having a population between 10,000 and 50,000 people. Micropolitan areas represent a rich and understudied unit of analysis [8,9] and, though accounting for only about 10% of U.S. population, comprise almost 60% of the country’s statistical urban units. The nature of these small but highly heterogeneous urban areas, and the place they hold between dense urban areas and rural areas, means they offer a unique opportunity to better understand both urbanization and deurbanization processes [10,11]. Differences in wages premiums may contribute to dynamics between metropolitan and micropolitan areas and so understanding how they differ between the two groups is important.

While many studies seek to identify the causes of an urban wage premium [12–20], our goal instead is to determine whether the wage premium predicted in [7] exists among micropolitan areas and, if so, how it compares to the wage premium of metropolitan areas. We also seek to examine how any differences between the two have changed over time. To answer these questions, we quantify the effect of city size on total wages for both metropolitan and micropolitan statistical areas for the period 1984 to 2020.

To quantify annual wage premiums for both metropolitan and micropolitan areas, we adopt a power-law function relating an area’s total annual wages to the area’s average annual employment, taking the power-law’s exponent *β* as the magnitude of the wage premium. A power-law relationship is superlinear if *β* > 1 and sublinear if *β* < 1. If *β* does not differ significantly from 1, the relationship to size is linear, meaning that the variable of interest is essentially independent of city size.

Power-law functions have been shown to describe relationships between city size and several social and economic attributes of cities [21,22], including the relationship between wages and city size for U.S. metropolitan statistical areas [5]. However, ours is the first study, to our knowledge, to extend this analysis to U.S. micropolitan statistical areas. Furthermore, our study makes an important contribution by analyzing wage scaling of U.S. urban areas over a 37-year period (see [6] for a 23-year study of wage scaling among Swedish cities).

In an attempt to explain these various urban scaling phenomena, Bettencourt [7] proposed a theoretical model that not only predicts the existence of an urban wage premium but calculates that its value should be approximately *β* = 7/6. Thus, we expected our study to find that an urban wage premium exists in both metropolitan and micropolitan areas and that it manifests as a power-law relationship between total wages and employment.

## Data and methods

Our spatial units of analysis are 926 core-based statistical areas (CBSAs) of the United States, covering all 50 states and the District of Columbia. This excludes 12 CBSAs of Puerto Rico that have lower quality data in the earlier years of our study. CBSAs are aggregations of one or more counties forming a coherent labor market and exhibiting a high degree of social and economic integration, particularly in terms of commuting patterns [23]. CBSAs are divided into two groups depending on population size. CBSAs with more than 50,000 residents are classified as metropolitan statistical areas (N = 384) while those having between 10,000 and 50,000 residents are classified as micropolitan statistical areas (N = 542).

We analyzed two possible measures of city size–population and employment. However, we found data on employment to be better matched to data on wages and thus use employment throughout this study as our measure of city size (see Supplemental Information for comparisons of results using population and discussion of its disadvantages).

Several federal agencies publish regional data on U.S. wages and employment, including the Bureau of Labor Statistics, the Bureau of Economic Analysis, and the Census Bureau. We evaluated each of these data sources and use data from the Bureau of Labor Statistics throughout this paper (see Supplemental Information for a comparison of results using alternate data sources).

Annual wage and employment data are taken from the U.S. Bureau of Labor Statistic’s Census of Employment and Wages (CEW) for the years 1984–2020 [24]. While CEW data is available for some years before 1984, we found that data to be of lesser quality and thus begin our analysis with 1984 data. The CEW includes wage and employment data for multiple geographic aggregations including counties, metropolitan statistical areas, states, and the U.S. in total. Here we use county-level data for three reasons. First, data are published for metropolitan statistical areas but not micropolitan statistical areas. However, because micropolitan statistical areas are aggregations of counties, we may construct micropolitan data from county data. Second, the designation of micropolitan statistical area, as currently defined, did not go into effect until 2003 (see [25] for a discussion of older attempts to define micropolitan areas). Yet, by using historical data on the counties they now comprise, we may examine their economic evolution before micropolitan areas existed as a separate CBSA category. Third, while changes to U.S. county boundaries are quite rare, the definitions of CBSAs, in terms of which counties they include, change periodically. This prevents meaningful comparisons over time and the U.S. Census Bureau suggests using a fixed delineation for such studies [26].

We aggregate county-level wage and employment data to core-based statistical areas using the September 2018 county-to-CBSA delineations published by the U.S. Office of Management and Budget [27]. Because we use a current county-to-CBSA mapping, we include CBSAs that may not have been designated as such at some point during our study period, for instance because they did not yet have the required population. (See Supplemental Information for other issues related to our decision to use a consistent delineation for all years of this study).

Finally, for both metropolitan and micropolitan areas, we adopt a power-law function to represent the relationship between a city’s total wages *W* in a given year and the city’s average employment *L* during that year

$$W_{i}=\alpha L_{i}^{\beta },$$ (1)

where *i* represents a given urban area and *β* is the power-law exponent which we take as the magnitude of a wage premium for a given year and category of urban area.

## Results

**Fig 1:**
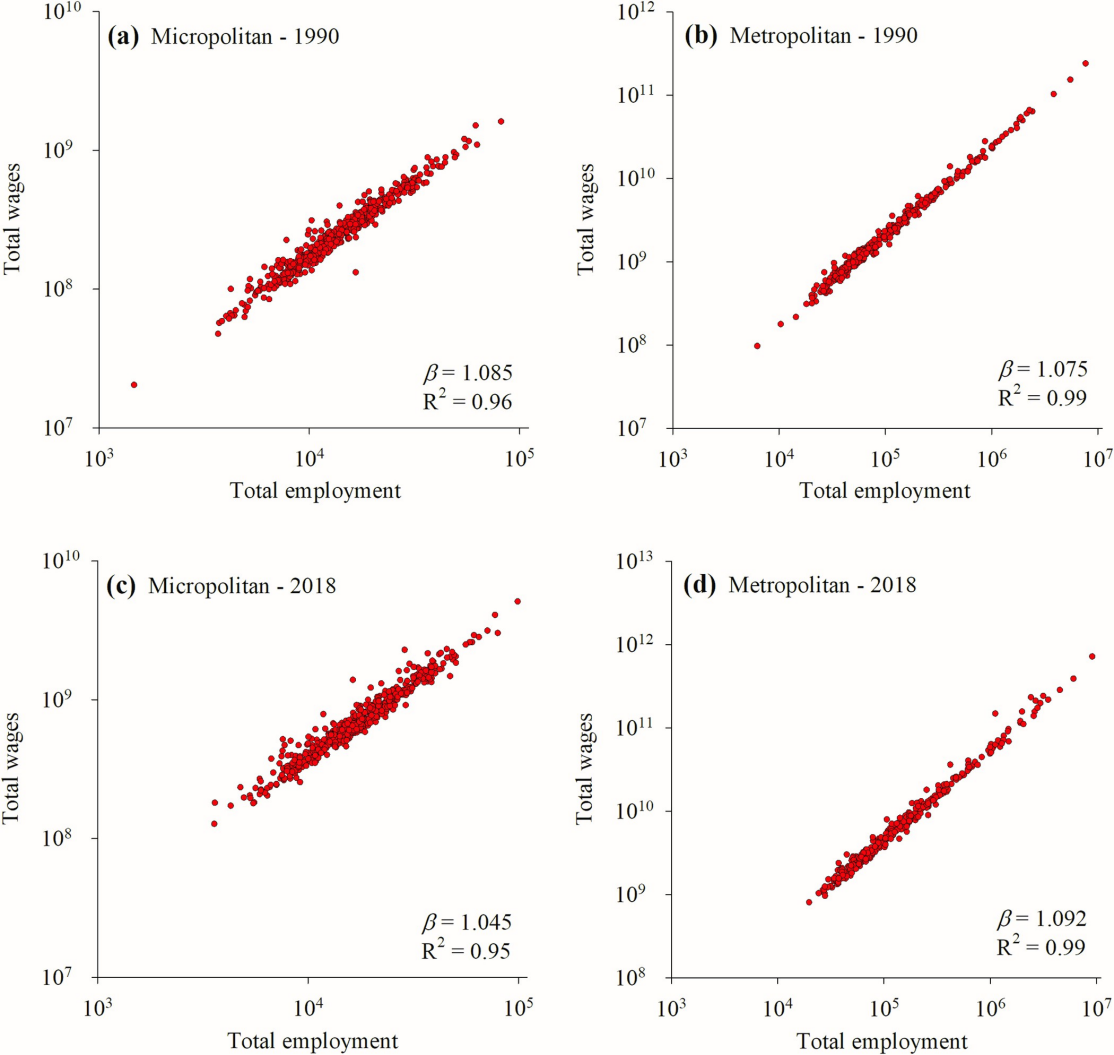
Scaling of total wages versus total employment. Power law regressions for four sample data sets are presented: Metropolitan and micropolitan areas for 1990 and 2018. The scaling coefficients determined in each regression correspond to single data points in Fig 2.

For each year 1984–2020 we analyzed two datasets, one for micropolitan statistical areas and one for metropolitan statistical areas, for a total of 74 annual datasets. For each set we calculated a power law scaling coefficient for total wages versus total employment. We take this scaling coefficient to be a measure of the strength of the urban wage premium across a given type of urban area. In all cases *p* < 10^−10^ and R^2^ > 0.94. Example plots of individual years for both metropolitan and micropolitan areas are shown in Fig 1.

**Fig 2:**
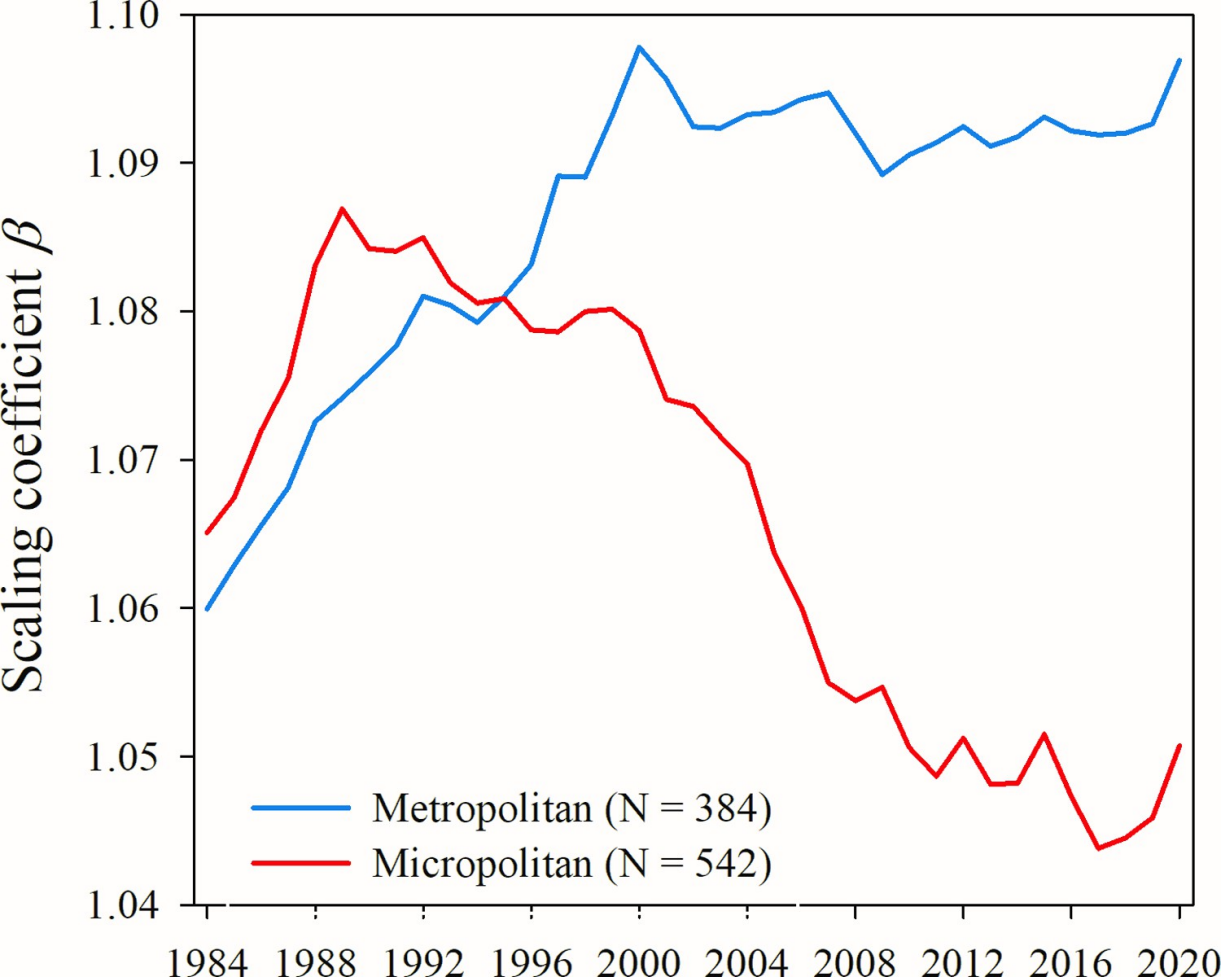
Metropolitan vs. micropolitan wage premium 1984–2020. We take the power function exponent *β* of log(wages) vs. log(employment) to be a measure of the urban wage premium for each area type. Wages here are not adjusted for regional differences in cost of living. See Supplemental Information for full table of results with confidence intervals.

Results in Fig 2 show that a wage premium does indeed exist for both micropolitan and metropolitan areas in all years of our study. However, we find that the magnitude of that premium is lower than the theoretically expected value of *β* = 7/6. Furthermore, we find that while the micropolitan wage premium increased throughout the 1980s and peaked in 1989, it has decreased since that time, steadily diverging both from the theoretically predicted value and from the value in metropolitan areas. The wage premium among micropolitan areas experienced a particularly rapid decline between 2000 and 2008 and has since continued to fall at a slower rate.

During the same time period the wage premium among metropolitan areas increased steadily through the 1980s and 1990s before peaking in 2000 and has since been relatively stable (Fig 2). Yet its magnitude has consistently below the value of *β* = 7/6 predicted theoretically in [7].

## Discussion

Overall, results suggests that opportunities for upward economic mobility have decreased in micropolitan areas over the past 37 years and the likelihood that an urban area will increase average wages by increasing size is now limited primarily to large cities. Several mechanisms may be underlying this divergence. In the following we discuss possibilities.

### Increasing economic complexity

To explain the diverging wage premiums between micropolitan and metropolitan areas, we focus on the notion that as cities grow in size they also grow in economic complexity [28–30] and interconnectedness [31]. This phenomenon was described qualitatively by Jacobs [32] and alluded to by Marshall [33] as early as 1920. One manifestation of increasing complexity is the appearance of new occupations. Indeed, the number of unique occupations in a city is correlated with the city’s total employment [34]. Often as a result of emerging technologies and innovations, some occupations that emerge as a city increases in size are occupations that did not previously exist anywhere. These novel occupations typically require highly specialized skill sets and the ability of these new jobs to appear is dependent on the pre-existence of required complimentary occupations and skills in the local economy [35]. This leads to the emergence of an industry hierarchy, based on city size, in which industries that depend on highly specialized skill sets, such as management, professional, scientific, and technology firms, emerge only if so-called primary industries are first present [22]. Thus, as the pace of technological innovation has increased in the last 37 years, metropolitan areas, with their larger size, have tended to be become the primary sites of growth in new high-skilled jobs.

To better understand how evolving differences between metropolitan and micropolitan areas relate to high-skill industries, we explore in more detail a sector that has experienced significant change during the study period, the information sector (NAICS code 51). The information sector is characterized by firms that (a) produce and distribute information and cultural products, (b) provide the means to transmit or distribute these products as well as data or communications, and (c) process data. Here we compare information sector employment and wages of 1990 (the first year NAICS-coded data is available) to those of 2018 for both metropolitan and micropolitan areas. Wages were adjusted for inflation and are stated in 2001 dollars (Table 1).

**Table 1:** Wage and employment changes in industry sector 51: Information Services, 1990–2018.

Area type	Year	Employment	Average Wages
Metropolitan	1990	2,560,249	43,936
	2018	2,618,102	81,365
	change	+ 2%	+ 85%
			
Micropolitan	1990	136,416	28,056
	2018	95,429	32,235
	Change	− 30%	+ 15%
			
U.S. total	1990	2,755,219	42,727
	2018	2,896,398	78,508
	change	+ 5%	+ 84%

Compiled from [24]. Wages are adjusted for inflation using [36] and stated in 2001 dollars.

Average annual inflation-adjusted wages in this industry experienced the largest percentage growth of any sector of the U.S. economy from 1990 to 2018, increasing from $42,757 in 1990 to $78,508 in 2018. However, this phenomenal growth in wages was not shared equally by metropolitan and micropolitan areas. While average information sector wages increased 85% in metropolitan areas between 1990 and 2018, they increased only 15% in micropolitan areas during the same period, a growth of less than one fifth.

Perhaps even more indicative of the growing divide between metropolitan and micropolitan areas, total information sector employment has increased in metropolitan areas by 2% since 1990 while decreasing by more than 30% in micropolitan areas. Thus, the last three decades has seen demand for high wages jobs in this lucrative industry sector shift away from micropolitan to metropolitan areas.

Other industries that experienced significant wage growth since 1990, including finance/ insurance and management of companies, also grew substantially more in metropolitan areas compared to micropolitan areas. On the other hand, industries which had the lowest wage increases since 1990, including retail trade and transportation/warehouse, had higher wage growth rates in micropolitan areas than metropolitan areas. A full analysis comparing all industry sectors 1990 to 2018 is presented in the Supplemental Information.

### The great divergence

While looking more deeply into individual industries may help illuminate the drivers of our result, it is also important to examine the broader socio-economic context in which the divergence in wage premiums takes place. Indeed, the divergence in wage premiums is but one of several attributes which has seen a divergence between smaller and larger cities. Following a sharp drop globally in income inequality following World War II, inequality again began to rise in the 1980s and 1990s [37,38]. This phenomenon, often referred to informally as the great divergence, has also been implicated in the divergence of wages and opportunity between larger and smaller U.S. cities [39,40]. Thus, the result we find may also be a manifestation of this broader divergence.

Explanations for this great divergence include the emergence of computer technology, which disrupted the skillsets demanded by workers and favored larger cities with their diverse pool of both novel skills and educational opportunities [39,40]. Thus, it is likely that the drivers of these broader trends are contributing to the divergence in wage premiums shown in Fig 2.

Another frequently cited driver of the great divergence is globalization, which has generally been shown to benefit large global cities to the exclusion of smaller ones [41–47]. Anecdotally, our results support this view, though we lack the data from before the current era of globalization to make a fuller evaluation.

We note that the onset of a decline in the micropolitan scaling coefficient coincides with a particular aspect of globalization which has been termed the 3^rd^ wave of globalization. This so-called 3^rd^ wave followed the fall of communism in Eastern Europe in 1989 and was marked by a global decrease in tariffs that reduced barriers to trade and altered international economic dynamics [48–50]. This period coincides with the onset of declining wage premiums in micropolitan areas and by 1995, the year that the World Trade Organization (WTO) came into existence, the wage premium for micropolitan areas fell below that of metropolitan areas for the first time in our study period. The micropolitan wage premium has continued to fall since that time.

The metropolitan wage premium, on the other hand, continued to increase through the 3rd wave of globalization, through the launch of the WTO, and through the late 1990s. Growth in the metropolitan wage premium only stopped after 2000 as the U.S. economy experienced the detrimental effects of both the Asian financial crisis of the late 1990s and the September 2001 terrorist attacks.

Finally, we note that both the halt in growth of the metropolitan wage premium as well as the onset of a period of rapid decline in the micropolitan wage premium (see Fig 1) coincide with another significant event in the process of globalization, China’s 2001 admission to the WTO. While we can infer no causal relationships between China’s admission and changes in U.S. urban wage premiums using the data in this study, we acknowledge that it represents an important question for future research.

### Deviation from theoretical prediction

Recall that Bettencourt [7] created an elegant theoretical model predicting not only the existence of a wage premium but that its value, measured as the scaling coefficient of wages versus city size, should be approximately 7/6 (~1.19). Yet, our are empirically derived scaling coefficients for metropolitan areas has been consistently lower in the past 20 years while those for micropolitan areas are not only lower but have been diverging from the theoretical prediction for 30 years. Bettencourt’s prediction was based on the understanding that social-economic phenomena are facilitated through social connectivity and interactions but constrained by features such as physical infrastructure. Thus, our result likely indicates that barriers to knowledge spillovers are preventing the wage premium from achieving is expected theoretical value. Such barriers might include the changes in the social dimension of work, for instance due to the growing prevalence of remote working. Given the growing divergence between empirical results and theoretical prediction, Bettencourt’s framework can guide future studies seeking to account for our observed values and temporal dynamics.

### Limitations of our study

The urban wage premium is often interpreted as an opportunity for workers to earn more in larger cities. Yet, previous studies are typically based on nominal wages and salaries. It remains unclear how urban wage premiums are affected when wages are adjusted for regional price differences. Using the Regional Price Parity Index (RPPI) for all items, published by the U.S. Bureau of Economic Analysis [51], we find the local cost of living index among metropolitan statistical areas is positively correlated with log(total employment) (R^2^ = 0.22, *p* < 0.001, data for 2019). This suggests that the scaling coefficient we take as a measure of the urban wage premium may be due, in part, to higher prices in larger areas. Therefore, further investigation of this effect is warranted [52].

Ideally, we would rerun our original analysis using the RPPI to adjust all wages for local price levels. Unfortunately, the RPPI includes local price levels only for metropolitan statistical areas and only for the years 2008–2019. Without similar price indices for micropolitan areas it is not possible to completely perform our analysis with adjusted wages. However, using what RPPI data is available we may at least probe the effects that price differences might have on our result.

**Fig 3:**
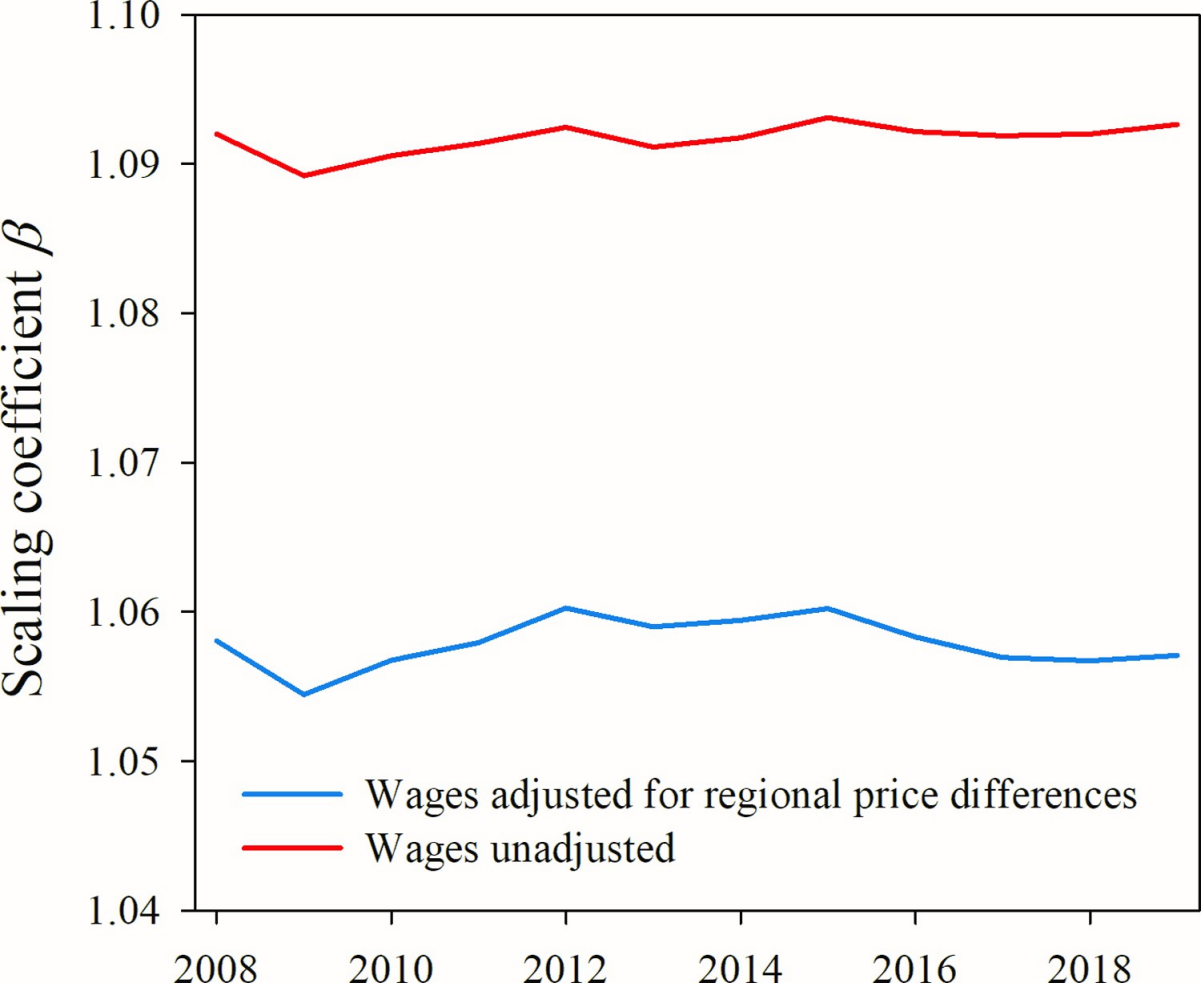
Cost of living effect on the metropolitan urban wage premium. When adjusted for local cost of living, scaling coefficients of log(wages) vs. log(employment) is suppressed by an average of 0.034 in all years compared. Cost of living factors, taken from [20], were published only for metropolitan statistical areas and only for years 2008–2019 at the time of our study.

To do so, we use the RPPI to adjust the subset of our data for which local price levels are available (Fig 3) and find that, while a wage premium among metropolitan statistical areas still exists when wages are adjusted for regional price differences, its magnitude is suppressed by approximately the same amount in all years. Thus, adjusting wages for local prices decreases the magnitude of the wage premium but does not qualitatively alter its trajectory across time.

What might this mean for micropolitan statistical areas? Faced with a similar lack of price data in Germany, Blien at al [53] created their own price indices for rural regions of Germany and found that differences in wages between urban and rural regions of Germany were minor when adjusted for regional cost of living. A similar study of counties in the U.S. state of Pennsylvania found that cost of living is significantly less in rural counties compared to urban counties [54] suggesting that the correlation we find between local price level and area size holds for smaller areas. Thus, when adjusted for regional prices, the micropolitan wage premiums presented in Fig 2 would likely be suppressed as were metropolitan premiums in Fig 3. It is even possible that *β* would be reduced to 1 meaning that a wage premium among micropolitan areas would no longer exist when prices are taken into account, though this could not be confirmed without actual price indices for micropolitan areas.

Taken together these studies and our limited exploration of the effect of price differences suggest that, while the premiums we measure with nominal wages are likely higher in magnitude than with real wages, it does not alter the fact that the premiums between metropolitan and micropolitan areas have been diverging for several years.

### Future directions

#### Linking results to other social-political trends

The goal of this study was to determine whether a wage premium exists among micropolitan areas and, if so, how it differs from that in metropolitan areas. One of our objectives in addressing this question is the hope that it may enrich the study of other diverging social, political, and cultural attributes between metropolitan and micropolitan areas. As an example of such potential contributions of our research we link our results to another growing difference between metropolitan and micropolitan areas, voter behavior. Using county level election returns from all presidential elections 1984–2020 [55–57], we aggregate vote totals for both metropolitan and micropolitan areas and compare results with our annual scaling of wages for each CBSA type.

**Fig 4:**
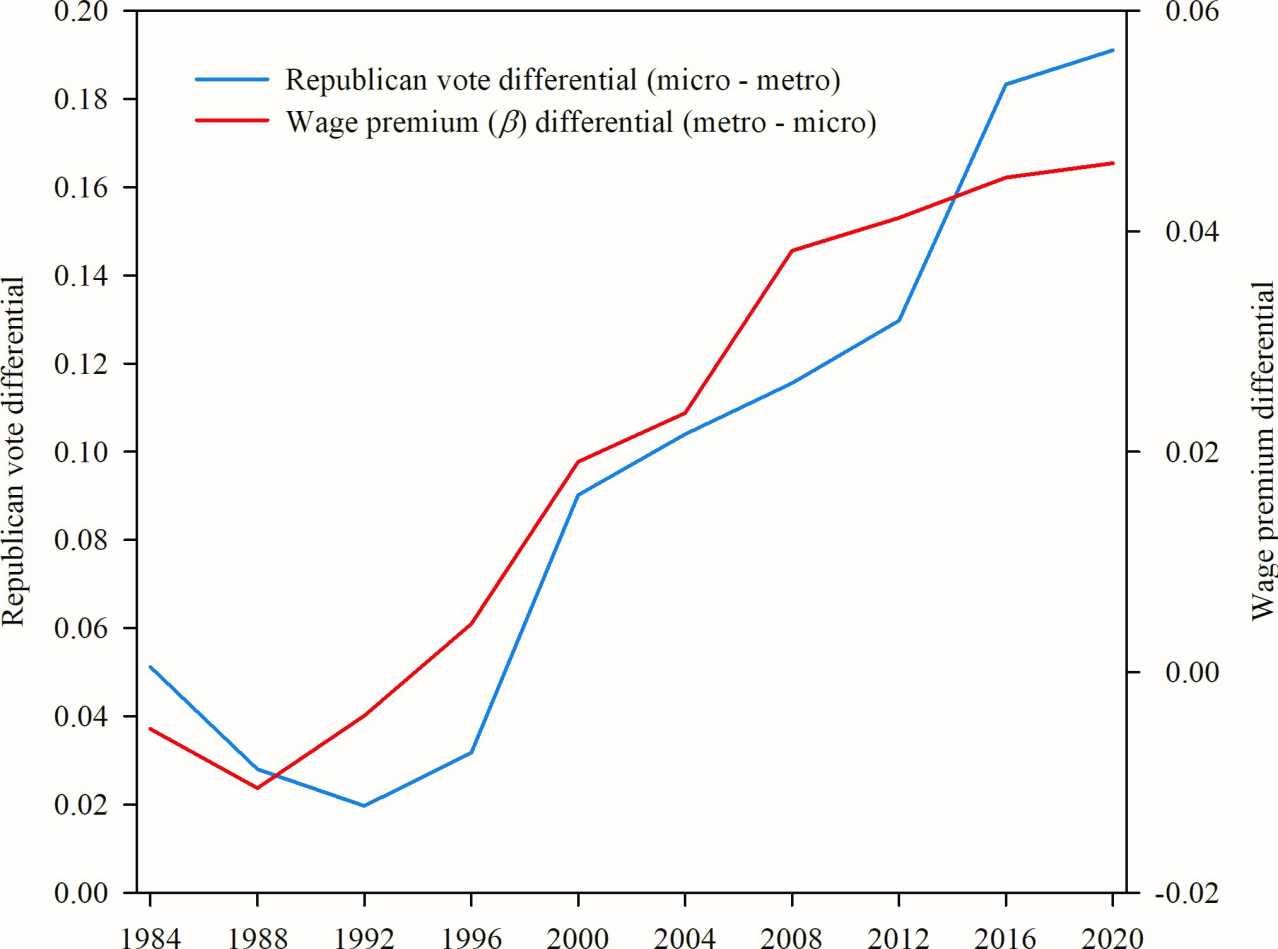
Metropolitan-micropolitan divergence of wage premium and presidential voting behavior. Here we plot only the differences between two attributes of metropolitan and micropolitan areas during presidential election years 1984–2020. The red line plots the metropolitan scaling coefficients minus the micropolitan scaling coefficients from Fig 1. The blue line plots, for presidential elections, the percentage of total votes in micropolitan areas that were for the Republican candidate minus the same percentage in metropolitan areas. As the gap between metropolitan and micropolitan wage premiums has increased since the late 1990s, so too has the difference in percent of votes for the Republican presidential candidate (N = 10, R2 = 0.91, p < 0.001).

Results are presented in Fig 4. In the 1992 U.S. presidential election, the percentage of votes for the Republican candidate was 1.9% higher in micropolitan areas than in metropolitan areas. This higher preference for conservative candidates in micropolitan areas has grown every year until, by the 2020 presidential election, the percentage of Republican votes in micropolitan areas was 19.5% higher than in metropolitan areas. Thus, as the gap in urban wage premiums has grown between metropolitan and micropolitan areas, a divergence in political preference has grown in close parallel (R2 = 0.91, p < 0.001).

It is important to note that we make no conclusion as to the causality of this trend but wish only to illustrate the contribution our analysis can make to better understanding a variety of diverging social, political, cultural, and economic attributes between U.S. metropolitan and micropolitan areas.

#### COVID-19 implications

Given the ongoing COVID-19 crisis at the time of this article’s writing, we feel it prudent to comment on the how the pandemic might impact the study’s main result. The pandemic has already significantly disrupted migration dynamics within the U.S., with 2021 having the lowest mover rate in over 70 years [58]. Though it is perhaps too recent to have made a significant impact in the scholarly literature, several media pieces speculate that the pandemic may actually reverse the decades-old trend of migration to cities [59–62]. These pieces generally suggest that a significant number of workers, particularly those of adequate financial means, will migrate from cities to places with less population density, including micropolitan or rural areas.

If these predictions prove generally accurate it is possible that the trends shown in Fig 2 will change significantly, if not reverse. It is also doubtful that the recipient areas of this anticipated exodus will be prepared for an influx of new residents, creating a disruption to the contemporary social, cultural, and political dynamics of smaller cities and towns across the U.S. Thus, future research in this area should analyze how migration of urban workers changes in response to the COVID-19 pandemic and what impact these changes have on both urban and rural economies.

#### Micropolitan cost of living

Finally, we re-emphasize the importance of analyzing the wage premium across micropolitan areas using price-adjusted wages. While our results show that a nominal wage premium does exist among micropolitan areas, the question of whether it continues to exist when wages are adjusted for regional price differences remains to be answered. This will require a systematic calculation of cost of living factors for U.S. micropolitan areas and thus, is a tantalizing direction for future research.

While not specifically addressing the wage premium, Korpi and Malmberg studied the difference between larger and smaller urban areas in Sweden by adjusting disposable income for differences in regional prices. They found that when disposable income was adjusted for general prices, income was positively related to population size [63], but when adjusted only for housing costs, real disposable was negatively related to population size. Thus, even if regional price parity estimates were created for U.S. micropolitan areas, it is likely that individual components of those estimates would greatly affect results of a wage premium analysis.

## Supporting information

S1 File (PDF)
