# Supplementary material for: The urban wage premium is disappearing in U.S. micropolitan areas
Source: PLoS One. 2022 Apr 14;17(4):e0267210. doi: 10.1371/journal.pone.0267210 (PMC9009609; doi:10.1371/journal.pone.0267210)
Supplement: S1 File — (PDF) [file pone.0267210.s001.pdf]

Supplementary Materials for

The Urban Wage Premium is Disappearing in U.S. Micropolitan Areas

Shade T. Shutters[1,2,*] and J. M. Applegate[1,2]

[1] School of Complex Adaptive Systems, Arizona State University, Tempe, Arizona 85282 USA

[2] Global Climate Forum, 10178 Berlin, Germany

*To whom correspondence should be addressed; shade.shutters@asu.edu.

This PDF file includes:

Section S1. Comparison of alternate employment and wage data sources

Section S2. Comparing alternate metrics of city size: total employment vs. population

Section S3. Sensitivity analysis for cutoff between metropolitan and micropolitan areas

Section S4. Full table of metropolitan and micropolitan scaling results 1984-2020

Section S5. Change in industry sector indicators, metropolitan areas, micropolitan areas, and total U.S., 1990-2018

Section S6. Scaling coefficients among non-CBSA counties 1984-2020

Figure S1. Scaling of wages versus employment using different data sources – by area type

Figure S2. Scaling of wages versus employment using different data sources – by data source

Figure S3. Scaling coefficients of total wages versus total employment and population, 1990-2020

Figure S4. Changing employment-to-population ratios in Regional Economic Accounts data of the Bureau of Labor Statistics.

Figure S5. Comparison of 2019 scaling coefficients for larger and smaller urban areas, under varying size boundaries

Figure S6. Scaling of total wages versus total employment for non-urban (non-CBSA) counties

Table S1. Scaling results for total wages versus total employment, 1984 – 2020

Table S2. Micropolitan and metropolitan wages and employment, 1990 and 2018, for all industry sectors

Supplemental References

Section S1. Comparison of alternate employment and wage data sources

In our study we used county-level employment and wage data published annually by the U.S. Bureau of Labor Statistics in its Census of Employment Wages (CEW) dataset [1]. These same categories of county-level data are also capture annually by the U.S. Census Bureau's County Business Patterns (CBP) dataset [2] and the U.S. Bureau of Economic Analysis' (BEA) Regional Economic Accounts data, specifically its dataset CAINC4-Personal Income and Employment by Major Component [3]. Each dataset has its advantages and drawbacks, primarily related to differences in coverage of government workers, non-profit businesses, and statistical augmentation [4]. We chose the CEW for our study because it is available for more years than the CBP and typically covers a larger number of employees than the other sets. Furthermore, the underlying wage data used by the BEA are based on place of work but the BEA attempts to adjust those values to reflect place of residence, making the wage and employment values published by the BEA inconsistent with regard to geography. We nevertheless take advantage of these alternative datasets to both validate our study's main finding and to present readers with a more comprehensive picture of metropolitan and micropolitan wage scaling.

The County Business Patterns data set is publicly available for download in data-ready format for years 1986 – 2019. Earlier years are available for download from the U.S. National Archives [5] but are not in a format that can be easily read by data analysis software. Thus, our analysis is limited to years 1986 and later.

The publicly available BEA Regional Economic Accounts data offers county-level wages, employment, and other measures, such as income, for the years 1969-2019. Thus, the BEA data offers a longer time period for temporal analysis but was not used in the main study for reasons highlighted above. Furthermore, the BEA data reports both total employment and wage and salary employment. Here we use wage and salary employment because it is meant to align with wages and salaries reported by the BEA. On the other hand, one could calculate a scaling coefficient for Total Employment (Wage and salary employment + Proprietors employment) versus (Wages and salaries + Proprietors' income), all of which are part of the BEA's CAINC4 dataset. However, that analysis is not performed here.

Using the methodology described in the main text we performed the same analysis on the CEW, CBP, and BEA datasets, comparing the scaling coefficients of metropolitan and micropolitan areas. Results shown both by area type (Figure S1) and by data type (Figure S2)

are similar using all data sources. With metropolitan areas, scaling coefficients using BLS and BEA data are nearly identical while coefficients derived with CBP data are slightly higher in all years (Figure S1). Temporal dynamics with all data sources follow similar dynamics. Micropolitan areas also show the same coarse trends, generally declining after 1990 with a slower rate of decline after 2010. However, micropolitan scaling coefficients in earlier years are considerably higher using CBP data but converge with CEW data over time. More importantly, all data sources show the same transition in the mid-to-late 1990s in which scaling coefficients in metropolitan areas become greater than those in micropolitan areas, followed by a sustained decline in micropolitan scaling coefficients.

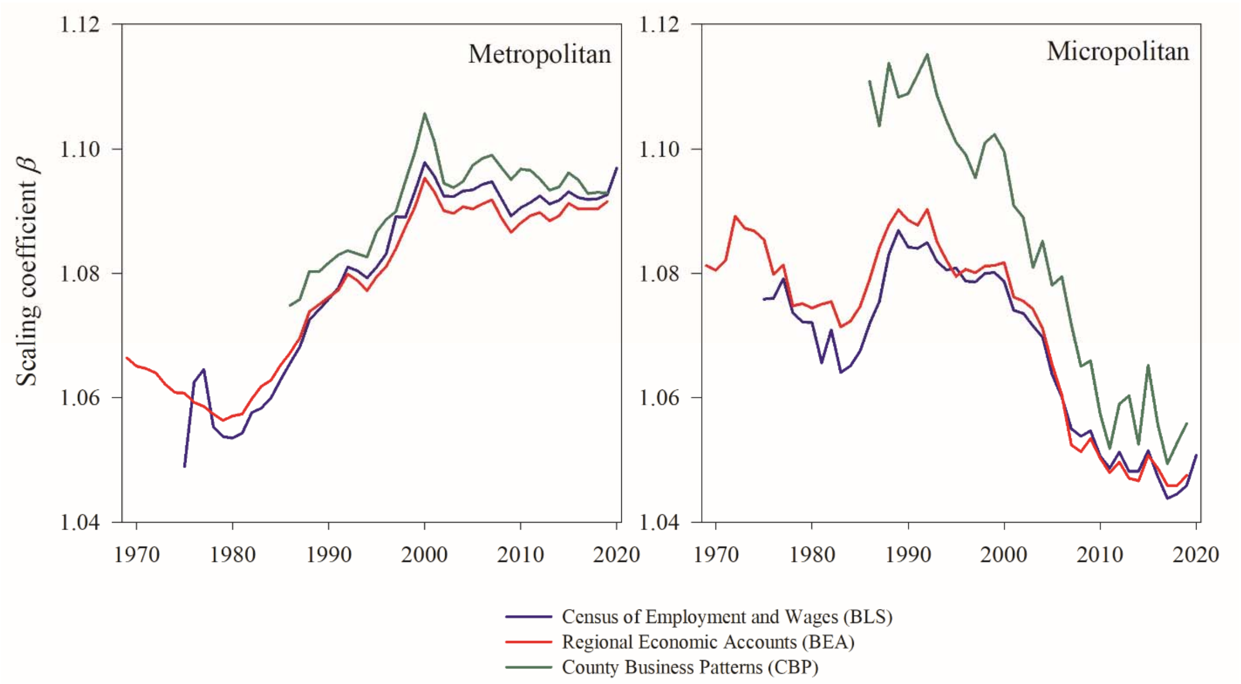

Figure S1. Scaling of wages versus employment using different data sources – by area type. Differences in scaling coefficients using different data sources for wages and employment are shown for (right) metropolitan areas and (left) micropolitan areas. CBP data results in higher scaling coefficients for all years in both area types, while scaling coefficients using CEW and BEA data are nearly identical. For all data types, area types, and years $R^2 > 0.94$.

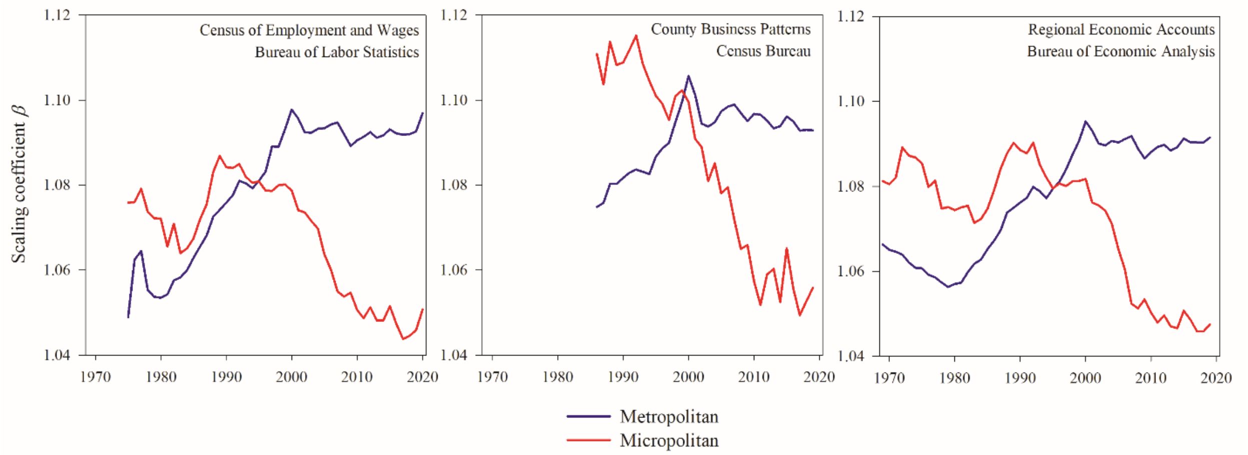

Figure S2. Scaling of wages versus employment using different data sources – by data source. Metropolitan and micropolitan scaling coefficients over time were calculated using the Census of Employment and Wages from the Bureau of Labor Statistics (left), County Business Patterns (CBP) from the Census Bureau (middle), and Regional Economic Accounts using Wage and Salary Employment from the Bureau of Economic Analysis (BEA) (right). Regardless of data source, a divergence is revealed between metropolitan and micropolitan scaling coefficients, which was preceded by a period in which micropolitan scaling coefficients were higher than those for metropolitan areas.

Section S2. Comparing alternate metrics of city size: total employment vs. population

In our study we use total employment as our measure of a city's size in order to compute a wage scaling coefficient. However, we note that other studies have calculated similar scaling coefficients using population as the measure of city size [e.g. 6]. We chose total employment because the Census of Earning and Wages (CEW) collects both employment and wages from the same business survey program. Thus, when data is suppressed for privacy reasons, both wage and employment data are equally affected, making aggregations more comparable than if one were to use different data sources for wages and employment.

For comparative purposes we include here additional results using population as the metric of city size. We extracted county-level populations from the U.S. Census Bureau's Annual Population Estimates [7] for years 1990 – 2020, linking those values by year to the county-level wages from the CEW. We then apply the methodology described in the main text to aggregate county data to core-based statistical areas (CBSAs) and calculate a scaling coefficient for wages versus population.

We further take county-level wages and population from the Bureau of Economic Analysis' CAINC4 dataset (BEA) described in Section S1 and performed the same analysis.

A comparison of our main results using employment with supplemental results using population is presented in Figure S3. We find that the scaling coefficients for metropolitan areas are higher when using population as the measure of city size, suggesting a change in the employment-to-population ratios across city sizes. On the other hand, scaling coefficients for micropolitan areas are lower when using population versus employment, so much so that scaling of wages versus population has been sublinear ($\beta < 1$) since 2005. The lower values also suggest an inconsistent relationship between employment and population across city size.

We speculate that a primary reason for the observed differences is that employment numbers are based on place of employment while population is based on place of residence. Despite a BEA procedure to adjust its wage data that it represents wages based on place of residence, results using their data is remarkably similar to the BLS data based on place of employment. Thus, commuting patterns will lead to differences in employment-to-population ratios across cities. A decrease in micropolitan scaling coefficient when using population would indicate that larger micropolitan areas have a larger share of residents working outside the micropolitan boundary. This may be a typical economic trend of urban growth, with new

businesses emerging more rapidly beyond the border of a core-based statistical area. It may also indicate shortcomings in the way the micropolitan statistical areas are defined. In any regard, the determination of the ideal metric of city size remains an interesting topic for future research.

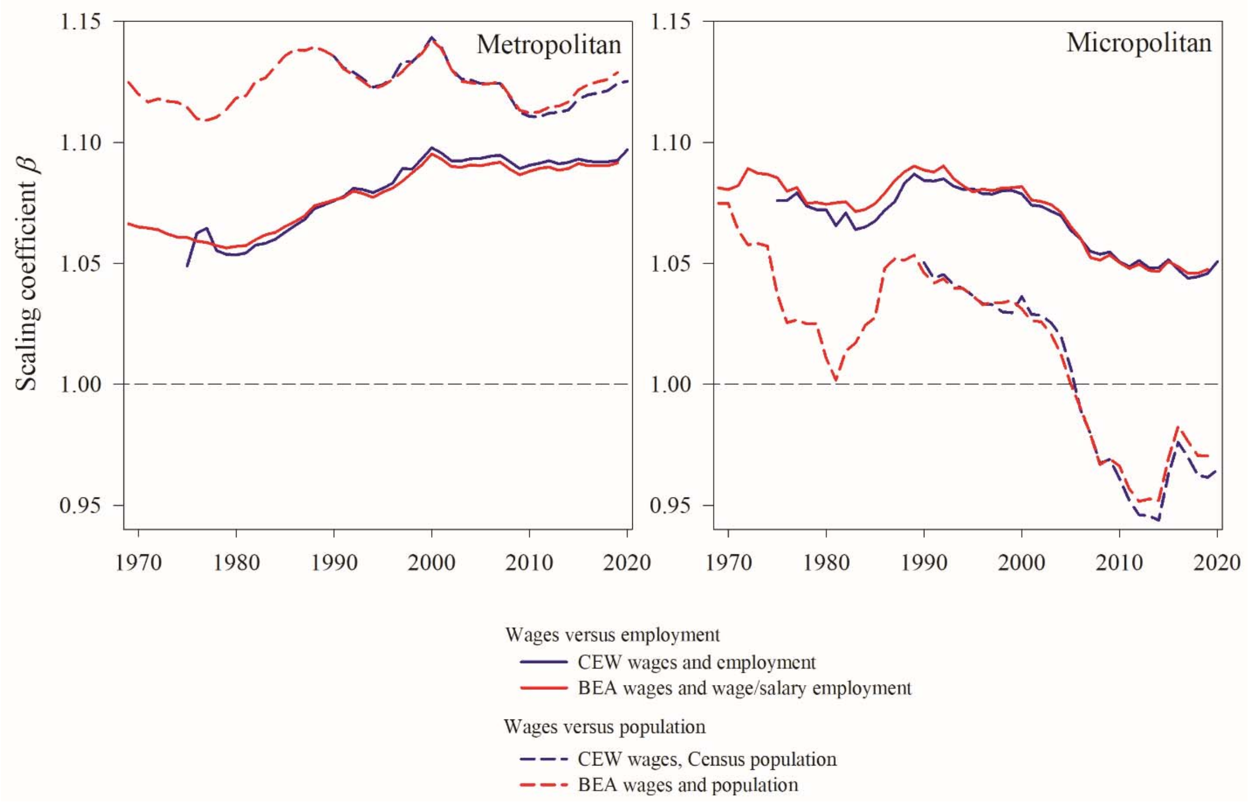

Figure S3. Comparison of scaling coefficients using population with coefficients using employment. Results are shown for both metropolitan (left) and micropolitan (right) areas. Using both BEA (red) and BLS (blue) data sources, scaling coefficients using population (dashed lines) are consistently higher for metropolitan areas and lower for micropolitan areas than coefficients using employment (solid lines). The relatively large gap between metro and micro population-based coefficients suggests that population is a less reliable measure of area size than employment. Dashed line at $\beta = 1$ indicates the boundary between superlinear ($\beta = 1$) and sublinear ($\beta < 1$) scaling.

Thus, our primary justification for use of employment over population as our measure of city size is that people represented in the employment data most closely match those in the wage data. However, because we did not know in detail the BEA's procedure for restating employment by place of residence, we sought to determine whether the ratio of employment to population is consistent over time. Therefore, we calculated the value of this ratio from 1969 to 2019 using annual population and annual total employment from the BEA Regional Economic Accounts CAINC4 dataset. For each year and each

CBSA type, we performed a linear regression of employment versus population generating a slope for each year and area type. We then plotted those slope values versus time to examine the stability of employment-to-population ratios and whether they differ between metropolitan and micropolitan areas.

The result presented in Figure S4 shows surprisingly that the ratio of employment-to-population is not only consistently higher in metropolitan areas, but the ratios of both CBSA types have steadily increased over the 51 years of available data. Neither the difference in CBSA types nor the ratio's systematic increase over time could be explained by periodic variations in unemployment rates. However, we note that the average population size of each category of CBSA also increased steadily over the period of our study and thus city size may influence the employment-to-population ratio. Further investigation into residential housing patterns, commute patterns, labor force participation rates, and data deficiencies related to, for instance, agricultural workers, may offer insights into the causes of this result.

For the purposes of this study, this inconsistency of employment-to-population ratios over time further justifies the use of employment as a measure of city size and not population.

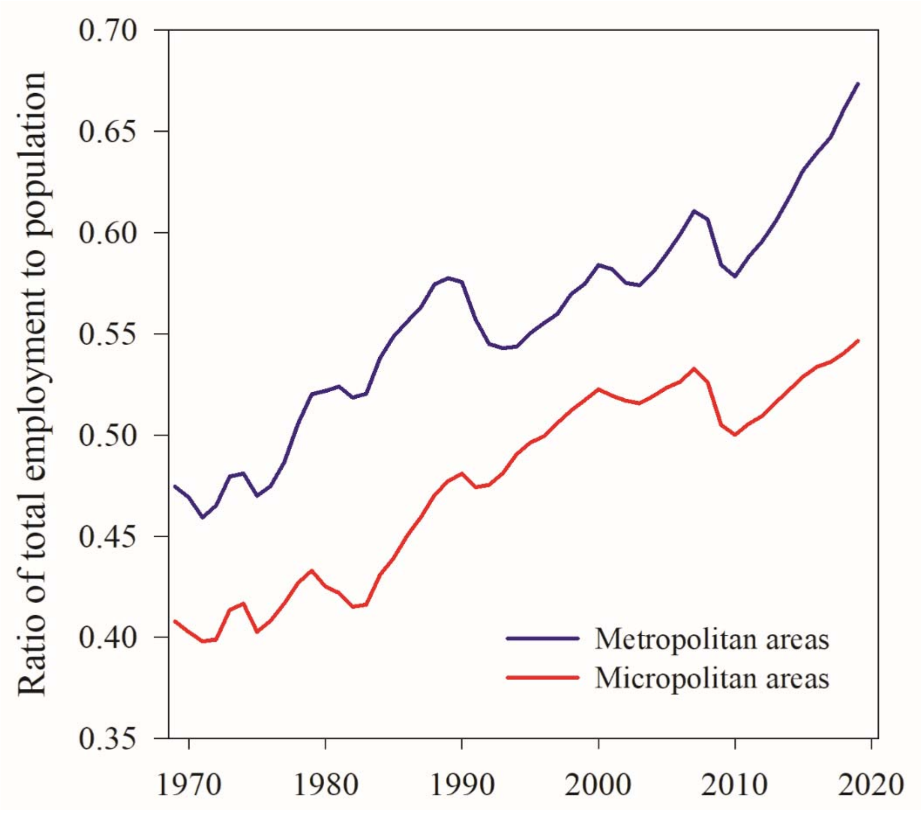

Figure S4. Changing employment-to-population ratios in Regional Economic Accounts data of the Bureau of Labor Statistics. The ratio of employment to population is the slope of a linear regression of employment versus population performed each year for each set of CBSA types. For all 102 regressions $p < 0.00001$.

Section S3. Sensitivity analysis of cutoff between metropolitan and micropolitan areas

A critical aspect of this study's design is the parsing of U.S. core-based statistical areas (CBSAs) into two mutual exclusive categories. We adopted the official demarcation used by all U.S. agencies, as designated U.S. Office of Management and Budget, of micropolitan and metropolitan statistical areas. The determination of category for any particular CBSA is based on a population threshold of 50,000, with CBSAs less than 50,000 designated as micropolitan and CBSAs more than 50,000 as metropolitan. We acknowledge that this threshold is seemingly arbitrary, yet adhere to the standard metro/micro schema for the following reasons:

- Any other cutoff used to distinguish larger cities from smaller cities would be equally arbitrary;
- The established convention of metropolitan and micropolitan areas has been used consistently across U.S. national agencies for decades and coincides with official government datasets;
- An existing body of literature on urban scaling going back at least as far as [6] has studied metropolitan areas as a distinct set of urban systems, and thus provides nearly 15 years of precedent upon which to build;
- Because certain government policies, including funding decisions, are based on the micro-metro determination, the very act of applying these labels can lead to qualitative differences between metropolitan and micropolitan areas [8].

Thus, we believe that, while arguably arbitrary, the cutoff adopted in the paper is superior to others.

Still, we feel it is important to explore the sensitivity of this cutoff value. To do so we further analyze 2019 data in the following manner. Instead of using the official government cutoff of 50,000 residents, we let the cutoff between large and small CBSAs vary of the range of 25,000 employees to 500,000 employees, in increments of 5,000 employees, and calculate the scaling coefficient for both large and small CBSA groups at each cutoff. The results in Figure S5, beta versus cutoff. We see almost no change in betas between large and small over this range of cutoffs, meaning that our cutoff falls within a highly stable range of otherwise arbitrary values and offers robust results.

Furthermore, to illustrate the degree of change in CBSA definitions across time we examined detailed changes between the 2003 CBSA definitions and 2018 definitions. We found that 74 CBSA codes that existed in 2003 had ceased to exist by 2018 while another 77 new CBSA codes had been added by 2018. These changes were due primarily to (a) mergers of or division of existing CBSAs, (b) substantial reconfiguration of the counties comprising a CBSA, or (c) new CBSAs emerging due to population growth.

Changes also included a switch in designation between micro or metro. Between 2003 and 2018, 28 micropolitan areas were redesignated as metropolitan areas while 2 metropolitan areas redefined a micropolitan areas. This is an average of less than 2 changes in designation per year out of nearly 940 CBSAs. Given all of these small but frequent changes in boundaries over time, the use of a consistent delineation schema for all years of data offers the best method for understanding temporal dynamics.

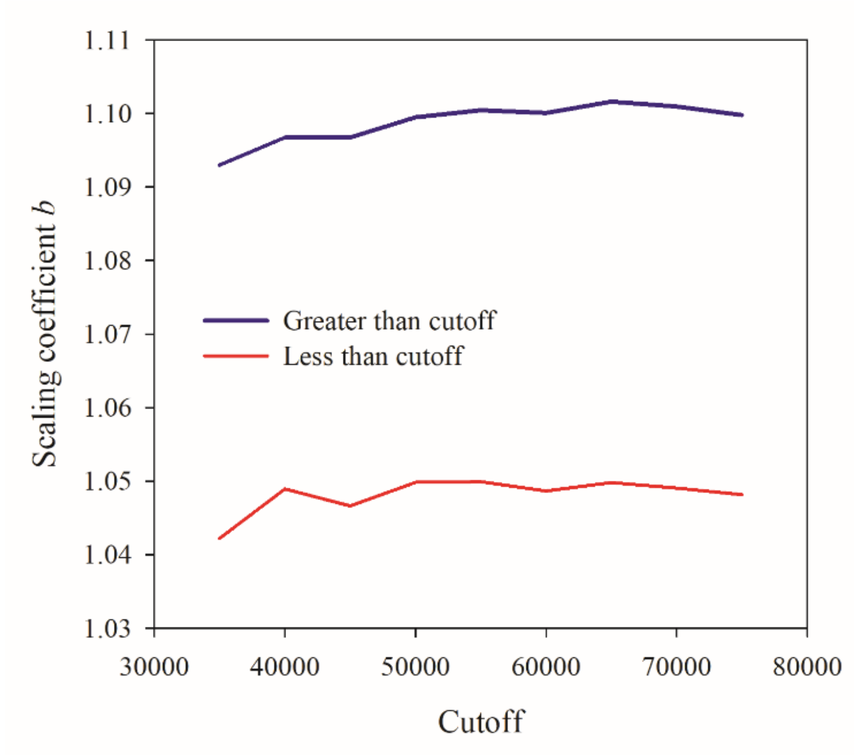

Figure S5. Comparison of 2019 scaling coefficients for larger and smaller urban areas, under varying size boundaries. Core-based statistical areas are classified as either small or large using a cutoff level of employment reported in the Census of Earnings and Wages (CEW). That cutoff is systematically analyzed between 35,000 and 75,000 in increments of 5,000 with a power-law scaling coefficient calculated for each group under each cutoff value. Results show little change over the range of analysis.

Section S4. Full table of metropolitan and micropolitan scaling results, 1984-2020

Here we present the full table of data represented graphically in Figure 2 of the main text. Values are derived from the Bureau of Labor Statistics' Census of Employment and Wages county-level data, aggregated to core-based statistical areas (CBSAs) using the September 2018 CBSA delineations published by the U.S. Office of Management and Budget.

Table S1. Scaling results for total wages versus total employment, 1984 – 2020*

	Metropolitan Areas (N = 384)			Micropolitan Areas (N = 542)		
**Year**	***β***	**95% C.I.**	**R2**	***β***	**95% C.I.**	**R2**
1984	1.060	± 0.010	0.991	1.065	± 0.020	0.951
1985	1.063	± 0.010	0.991	1.067	± 0.020	0.953
1986	1.066	± 0.010	0.992	1.072	± 0.019	0.957
1987	1.068	± 0.010	0.992	1.075	± 0.018	0.960
1988	1.073	± 0.010	0.992	1.083	± 0.019	0.960
1989	1.074	± 0.010	0.992	1.087	± 0.018	0.962
1990	1.076	± 0.010	0.992	1.084	± 0.019	0.959
1991	1.078	± 0.010	0.992	1.084	± 0.018	0.962
1992	1.081	± 0.010	0.992	1.085	± 0.018	0.961
1993	1.080	± 0.010	0.992	1.082	± 0.019	0.960
1994	1.079	± 0.010	0.992	1.081	± 0.018	0.961
1995	1.081	± 0.010	0.992	1.081	± 0.022	0.946
1996	1.083	± 0.010	0.992	1.079	± 0.018	0.963
1997	1.089	± 0.011	0.991	1.079	± 0.018	0.963
1998	1.089	± 0.010	0.991	1.080	± 0.018	0.964
1999	1.093	± 0.011	0.991	1.080	± 0.017	0.965
2000	1.098	± 0.011	0.990	1.079	± 0.018	0.964
2001	1.096	± 0.010	0.991	1.074	± 0.017	0.967
2002	1.092	± 0.010	0.992	1.074	± 0.017	0.967
2003	1.092	± 0.010	0.991	1.072	± 0.017	0.966
2004	1.093	± 0.010	0.991	1.070	± 0.017	0.965
2005	1.093	± 0.010	0.991	1.064	± 0.017	0.964
2006	1.094	± 0.010	0.991	1.060	± 0.018	0.961
2007	1.095	± 0.011	0.991	1.055	± 0.019	0.957
2008	1.092	± 0.011	0.991	1.054	± 0.020	0.954
2009	1.089	± 0.010	0.991	1.055	± 0.019	0.958
2010	1.091	± 0.011	0.991	1.051	± 0.019	0.954
2011	1.091	± 0.011	0.990	1.049	± 0.020	0.950
2012	1.092	± 0.011	0.990	1.051	± 0.021	0.946
2013	1.091	± 0.011	0.990	1.048	± 0.021	0.945
2014	1.092	± 0.011	0.989	1.048	± 0.022	0.942
2015	1.093	± 0.011	0.990	1.051	± 0.021	0.948
2016	1.092	± 0.011	0.990	1.047	± 0.019	0.954
2017	1.092	± 0.011	0.990	1.044	± 0.020	0.952
2018	1.092	± 0.011	0.990	1.045	± 0.020	0.949
2019	1.093	± 0.011	0.990	1.046	± 0.021	0.949
2020	1.097	± 0.012	0.990	1.051	± 0.020	0.953

* C.I. = confidence interval. $p < 10^{-10}$ in all cases.

Section S5. Change in industry sector indicators, metropolitan areas, micropolitan areas, and total U.S., 1990 – 2018

In the main text we present an in-depth look at the industry sector Information. Here we present the same in-depth analysis of all industry sectors (Table S1), which are defined as the 2-digit level of aggregation using the North American Industry Classification System (NAICS). NAICS codes were first used in the Bureau of Labor Statistics' Census of Earnings and Wages in 1990. Thus, we compare 1990 to the latest year available at the time of this detailed analysis.

Table S2. Micropolitan and metropolitan wages[1] and employment, 1990 and 2018, for all industry sectors

NAICS code: Industry Sector	Year	Metropolitan areas Workers	Metropolitan areas Average Wages	Micropolitan areas Workers	Micropolitan areas Average Wages	Total US Workers	Total US Average Wages
11: Agriculture, forestry, fishing and hunting	1990	99,835	18,906	6,211	21,079	622,247	18,961
	2018	725,205	24,800	101,485	26,423	944,534	25,058
	change	45.1%	31.2%	80.5%	25.3%	51.8%	32.2%
21: Mining, quarrying, and oil and gas extraction	1990	316,531	55,964	88,018	43,402	470,414	52,285
	2018	368,880	83,875	126,613	60,884	606,237	75,646
	change	16.5%	49.9%	43.8%	40.3%	28.9%	44.7%
22: Utilities	1990	396,125	52,043	30,994	44,675	444,715	50,778
	2018	489,184	74,786	51,595	60,317	570,546	72,509
	change	23.5%	43.7%	66.5%	35.0%	28.3%	42.8%
23: Construction	1990	4,396,298	36,583	344,616	28,680	4,961,656	35,485
	2018	6,325,344	44,914	450,524	35,986	7,199,935	44,009
	change	43.9%	22.8%	30.7%	25.5%	45.1%	24.0%
31-33: Manufacturing	1990	14,542,751	39,988	2,040,622	30,607	17,791,645	37,902
	2018	10,166,987	51,106	1,546,806	37,020	12,629,733	48,203
	change	-30.1%	27.8%	-24.2%	21.0%	-29.0%	27.2%
42: Wholesale trade	1990	4,308,286	42,574	230,134	27,924	4,691,879	41,276
	2018	4,707,823	53,696	256,854	38,288	5,618,143	55,173
	change	9.3%	26.1%	11.6%	37.1%	19.7%	33.7%
44-45: Retail trade	1990	11,516,066	21,601	1,151,036	17,192	13,388,825	20,891
	2018	13,755,864	23,226	1,290,594	19,248	15,909,625	22,711
	change	19.4%	7.5%	12.1%	12.0%	18.8%	8.7%
48-49: Transportation and warehousing	1990	3,102,273	38,390	182,494	31,815	3,409,033	37,720
	2018	5,059,533	39,184	350,376	33,651	5,681,149	38,680
	change	63.1%	2.1%	92.0%	5.8%	66.6%	2.5%
51: Information	1990	2,560,249	43,936	136,416	28,056	2,755,219	42,757
	2018	2,618,102	81,365	95,429	32,235	2,896,398	78,508
	change	2.3%	85.2%	-30.0%	14.9%	5.1%	83.6%
52: Finance and insurance	1990	4,630,355	43,447	235,885	27,753	4,989,958	42,263
	2018	5,318,707	79,955	247,731	40,328	5,889,621	76,960
	change	14.9%	84.0%	5.0%	45.3%	18.0%	82.1%
53: Real estate and rental and leasing	1990	1,589,783	28,882	75,577	18,496	1,696,595	28,196
	2018	2,047,517	42,520	106,956	27,063	2,228,757	41,434
	change	28.8%	47.2%	41.5%	46.3%	31.4%	46.9%
54: Professional and technical services	1990	4,515,116	48,594	120,785	32,913	4,699,596	47,875
	2018	8,319,056	69,876	228,739	42,996	9,247,984	68,613
	change	84.2%	43.8%	89.4%	30.6%	96.8%	43.3%
55: Management of companies and enterprises	1990	878,184	55,166	8,878	42,935	895,774	54,756
	2018	2,136,851	88,363	54,413	59,216	2,270,113	87,015
	change	143.3%	60.2%	512.9%	37.9%	153.4%	58.9%
56: Administrative and waste services	1990	4,161,035	21,790	128,052	19,644	4,339,937	21,659
	2018	7,977,683	29,073	383,560	22,193	9,229,165	28,826
	change	91.7%	33.4%	199.5%	13.0%	112.7%	33.1%
61: Educational services	1990	3,239,413	31,585	216,493	27,549	3,570,024	31,099
	2018	6,301,450	36,765	406,942	28,470	6,998,752	35,886
	change	94.5%	16.4%	88.0%	3.3%	96.0%	15.4%
62: Health care and social assistance	1990	8,495,888	33,043	434,346	25,755	9,145,130	32,350
	2018	18,191,709	36,995	1,163,724	30,988	19,953,859	36,443
	change	114.1%	12.0%	167.9%	20.3%	118.2%	12.7%
71: Arts, entertainment, and recreation	1990	1,407,707	20,866	100,636	13,716	1,540,738	20,281
	2018	2,285,476	27,903	143,267	16,668	2,508,522	27,269
	change	62.4%	33.7%	42.4%	21.5%	62.8%	34.5%
72: Accommodation and food services	1990	6,775,240	13,042	635,046	10,206	7,640,183	12,715
	2018	12,242,225	15,561	1,023,414	12,432	13,775,637	15,246
	change	80.7%	19.3%	61.2%	21.8%	80.3%	19.9%
81: Other services, except public administration	1990	3,073,563	21,987	232,257	15,671	3,417,768	21,337
	2018	4,021,807	27,565	271,230	20,496	4,479,723	27,007
	change	30.9%	25.4%	16.8%	30.8%	31.1%	26.6%
92: Public administration	1990	5,099,269	37,924	421,525	27,412	5,914,486	36,024
	2018	5,893,284	49,558	579,418	33,272	6,963,236	46,884
	change	15.6%	30.7%	37.5%	21.4%	17.7%	30.1%

[1]Wages are adjusted to 2001 dollars.

Section S6. Scaling coefficients among non-CBSA counties 1984-2020

While our primary goal was to determine the presence of an urban wage premium among micropolitan areas and how that differed over time compared to metropolitan areas, we also considered those counties that do not fall into a metropolitan or micropolitan statistical area. Because there is considerable variability in these counties, we used a classification schema published by the U.S. Department of Agriculture's Economic Research Service called the Rural Urban Continuum Codes (URCC). The latest version of this system was created in 2013 and is publicly available at [9]. This schema classifies all U.S. counties into one of nine types, four of which deal with counties that are not already part of a core-based statistical area (CBSA).

After removing all counties that are part of a CBSA using the Office of Management and Budget's September 2018 CBSA delineations, we used the URCC to classify remaining U.S. counties as either adjacent to an MSA or not and either less than or greater than 2,500 residents, resulting in four county groups. We then calculated scaling coefficients for total wages versus total employment for each of the four categories for the years 1984 to 2020 using the method described in the main text.

Results shown in Figure S6 indicate that, like both CBSA types, each rural county group exhibits a wage premium (unadjusted for local prices), meaning that workers in larger rural counties earn higher average wages than workers in smaller rural counties. Over time all categories of rural counties have followed a trajectory similar to that of micropolitan statistical areas, having declined since the 1990s, until they have all converged to scaling coefficients nearly equal to that of micropolitan areas.

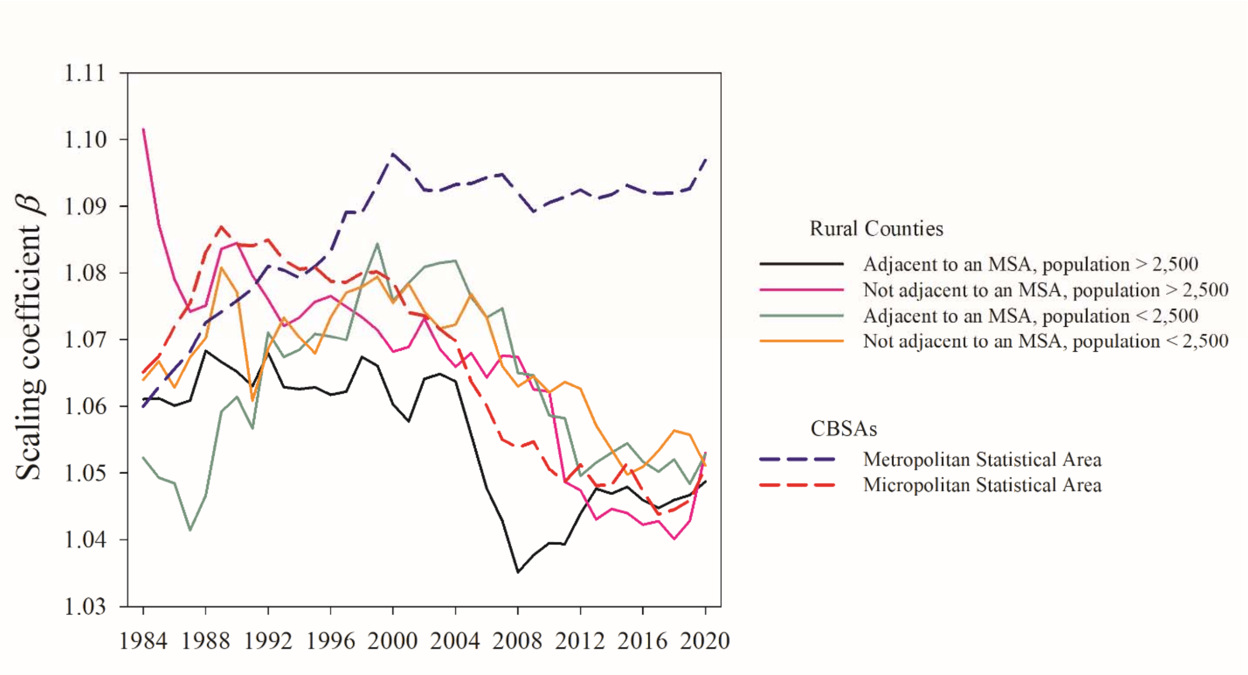

Figure S6. Scaling of total wages versus total employment for non-urban (non-CBSA) counties. Scaling for four categories of rural counties are shown along with metropolitan and micropolitan areas discussed in the main text. All categories of rural counties have historical trajectories which track that of micropolitan areas.

Supplemental References

1. U.S. Bureau of Labor Statistics (2021). *Quarterly Census of Employment and Wages*. Available at: https://www.bls.gov/cew/.
2. U.S. Census Bureau (2021). *County Business Patterns (CBP): Abount this program*. Available at: https://www.census.gov/programs-surveys/cbp/about.html.
3. U.S. Bureau of Economic Analysis (2021). *Personal Income by County and Metropolitan Area, 2019*. Available at: https://www.bea.gov/data/income-saving/personal-income-county-metro-and-other-areas.
4. U.S. Bureau of Economic Analysis (2020). *What is the difference between BEA employment and wages and BLS and Census employment and wages?* Available at: https://www.bea.gov/help/faq/104.
5. U.S. National Archives (2020). *County Business Patterns (CBP) Files, ca. 1974 - 2007*. Available at: https://catalog.archives.gov/id/613576.
6. Bettencourt, Luis M. A., José Lobo, Dirk Helbing, Christian Künhert, and Geoffrey B. West (2007). Growth, innovation, scaling, and the pace of life in cities. *Proceedings of the National Academy of Sciences*, 104(17):7301-7306.
7. U.S. Census Bureau (2021). *Population Estimates*. Available at: http://www.census.gov/popest/data/index.html.
8. Nemerever, Zoe and Melissa Rogers (2021). Measuring the Rural Continuum in Political Science. *Political Analysis*, 29(3):267-286.
9. U.S. Department of Agriculture: Economic Research Service (2020). *Rural-Urban Continuum Codes*. Available at https://www.ers.usda.gov/data-products/rural-urban-continuum-codes/.
